# In Vivo Evaluation of Praziquantel-Loaded Solid Lipid Nanoparticles against *S. mansoni* Infection in Preclinical Murine Models

**DOI:** 10.3390/ijms23169485

**Published:** 2022-08-22

**Authors:** Tayo A. Adekiya, Pradeep Kumar, Pierre P. D. Kondiah, Philemon Ubanako, Yahya E. Choonara

**Affiliations:** Wits Advanced Drug Delivery Platform Research Unit, Department of Pharmacy and Pharmacology, School of Therapeutic Sciences, Faculty of Health Sciences, University of the Witwatersrand, Johannesburg 2193, South Africa

**Keywords:** praziquantel, solid lipid nanoparticles, Compritol ATO 888, soy lecithin, *S. mansoni*, schistosomiasis, stability

## Abstract

This study aimed to develop and assess the long-term stability of drug-loaded solid lipid nanoparticles (SLNs). The SLNs were designed to extend the release profile, overcome the problems of bioavailability and solubility, investigate toxicity, and improve the antischistosomal efficacy of praziquantel. The aim was pursued using solvent injection co-homogenization techniques to fabricate SLNs in which Compritol ATO 888 and lecithin were used as lipids, and Pluronic F127 (PF127) was used as a stabilizer. The long-term stability effect of the PF127 as a stabilizer on the SLNs was evaluated. Dynamic light scattering (DLS) was used to determine the particle size, stability, and polydispersity. The morphology of the SLNs was examined through the use of transmission electron microscopy (TEM) and scanning electron microscopy (SEM). The chemical properties, as well as the mechanical, thermal, and crystal behaviours of SLNs were evaluated using FTIR, ElastoSens Bio2, XRPD, DSC, and TGA, respectively. SLNs with PF127 depicted an encapsulation efficiency of 71.63% and a drug loading capacity of 11.46%. The in vitro drug release study for SLNs with PF127 showed a cumulative release of 48.08% for the PZQ within 24 h, with a similar release profile for SLNs’ suspension after 120 days. DLS, ELS, and optical characterization and stability profiling data indicate that the addition of PF127 as the surfactants provided long-term stability for SLNs. In vitro cell viability and in vivo toxicity evaluation signify the safety of SLNs stabilized with PF127. In conclusion, the parasitological data showed that in *S. mansoni*-infected mice, a single (250 mg/kg) oral dosage of CLPF-SLNs greatly improved PZQ antischistosomal efficacy both two and four weeks post-infection. Thus, the fabricated CLPF-SLNs demonstrated significant efficiency inthe delivery of PZQ, and hence are a promising therapeutic strategy against schistosomiasis.

## 1. Introduction

Praziquantel is an anthelmintic drug employed to treat parasitic worm diseases, such as schistosomiasis. Praziquantel (PZQ) is the only available drug to treat schistosomiasis at the moment. Praziquantel acts by producing severe muscle spasms and paralysis in the worms. This paralysis is accompanied by and most likely caused by a fast Ca^2+^ influx inside the schistosome [1]. Even though PZQ is effective against adult worms, two major limitations associated with the use of the drug have been reported, which include rapid metabolism that decreases the drug’s bioavailability in circulation, and the drug’s minimal degree of efficacy against juvenile and immature forms of the *Schistosoma* worm [2]. More so, multidrug resistance against PZQ has been documented in some regions of the world [3].

However, no meaningful and considerable impetus has been given to the discovery of new drugs for the treatments of schistosomiasis, which may be due to a lack of motivation and poor financial support for pharmaceutical researchers in that research area [4]. Additionally, there is a huge expense associated with the process of developing new molecules or novel chemical entities (NCEs) through the long drug discovery pipeline [4]. Thus, pharmaceutical scientists have focused on improving the therapeutic efficacy of the gold-standard drug, PZQ, by developing more efficacious delivery systems through a nanomedicine approach. The approach uses nanotechnological-based techniques in advanced drug delivery systems (DDS) as well as targeted strategies in the improvement of pharmaceutical ingredients for treatments of diseases and disorders [5,6,7,8].

Hence, this study aims to improve the effectiveness of the drug by enhancing its bioavailability via reducing the release of PZQ over time and enhancing its effectiveness against the immature forms of the Schistosoma parasite. SLNs can overcome the problems of solubility and bioavailability faced by pharmaceutical ingredients with poor permeability [9,10,11]. SLNs can encapsulate drugs with different physiological and pharmacological characteristics [9,10,11]. Additionally, their multidrug resistance mechanisms’ bypass activities have been reported [12]. Meanwhile, lipid-based nanosystems have been shown to mimic the formation of chylomicrons, which gives this type of delivery system the ability to transport the entrapped or encapsulated bioactive molecules effectively when passing through the classical transcellular lipid absorption mechanism [5,6,7,8]. More so, for the treatment of schistosomiasis, it is believed that a lipid-based nanosystem will improve PZQ modification and target rate, hence enhancing and improving drug absorption past the schistosome’s biological barrier (tegument) in the human host.

The stability of pharmaceutical products influences the efficacy and safety of the product, as degradation impurities could result in a loss of efficacy and possibly generate side effects. Given this, the attainment of the physical and chemical stability of drugs is very important to ensure their quality and safety [13]. The application of surfactants as a stabilizer for carrier systems has been proposed as one of the easiest and most successful approaches for sustaining release profiles and improving the physicochemical characteristics of the nano-delivery system, as well as enhancing the chemical stability of entrapped lipid-soluble drugs [14]. Several other studies that have explored the use of PF127 to improve the stability and solubility of drug molecules include the study carried out by Shaarani et al [15], in which it was discovered that the addition of PF127 to the nanodispersion system maintained the stability of lutein for days of storage. The preparation of gold nanoparticles (GNP) through the addition of PF127 with chloroauric acid (HAuCl_4_) was carefully studied by Sokolsky-Papkov and Kabanov [16]. It was reported in their study that the presence of PF127 improves the methods of preparing pure, stable, and uniform GNP, which can be employed in nanomedicine. More so, the stability of the dispersion of nanocarriers in a colloidal system influences therapeutic formulation efficacy in terms of post-reconstitution in the withdrawal of the dose and before oral administration [17].

Thus, this study seeks to investigate the long-term stability of PZQ-loaded Compritol ATO 888-lecithin solid lipid nanoparticles solution stabilized with pluronic F127 (PF127) surfactant using dynamic light scattering (DLS), electrophoretic light scattering (ELS), the Turbiscananalyzer, and a transmission electron microscope (TEM). Furthermore, the study investigates the efficacy of the PZQ-loaded Compritol ATO 888-lecithin solid lipid nanoparticles solution stabilized with PF127 surfactant nanoformulation. The in vitro cell viability and in vivo toxicity evaluation were carried out to ascertain the safety of the nanoformulations. Additionally, the antischistosomal activities against *Schistosomamansoni* infection in a pre-clinical model were conducted to determine the parasitological cure rate.

## 2. Results

### 2.1. Physical Analysis of the Nanoparticulate System

**Figure 1:**
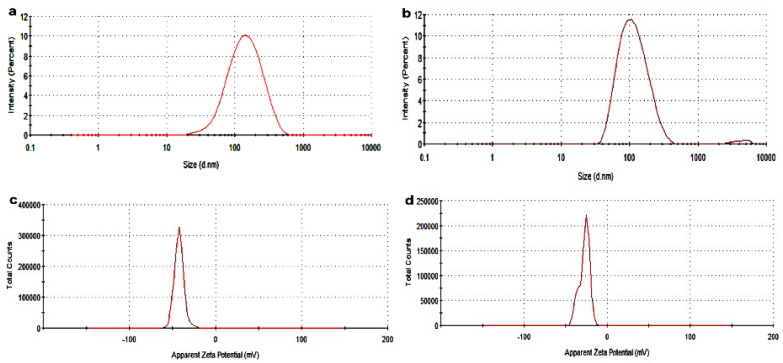
The generated particle size intensity profiles for (**a**) CLF and (**b**) CLPF. The particle zeta potential measurement profiles for (**c**) CLF (**d**) CLPF by dynamic light scattering. The samples were suspended in distilled water before measurement.

PZQ-loadedCompritol-lecithin-SLN (CLPF-SLN) and unloaded Compritol-lecithin SLN (CLF-SLN) were characterized. Table 1 shows the average particle size distribution, zeta potential, and PDI; these were determined after the suspension was dialyzed and washed using dialysis tubing with M.W. 12,000 Da. The generated average particle size distribution, as well as the zeta potential for the formulated SLNs, showed that there was uniformity in the size and zeta potential of the formulations, as shown in Figure 1. The statistical analysis showed no significant difference in the size of the particle distribution, PDI, and zeta potential of the SLNs. The particle sizes of CLF-SLN and CLPF-SLN were 101.6 ± 0.7 nm and 112.9 ± 1.0 nm, respectively. The size of particles is an essential factor that affects the stability, biodistribution, and release of the drug [18].

The polydispersity index of the formulations ranges between 0.23 to 0.27 a.u. which is an indication of the homogenous population of the SLN. The average zeta potential of the SLN formulations ranged between −19.0 ± 0.26 and −23.6 ± 0.26, which indicates that the formulations are stable in terms of aggregation, flocculation, sedimentation, coagulation, and creaming via a strong electrostatic repulsion that keeps the particles charged from one another. Meanwhile, any dispersions with a lesser zeta potential value will undergo inter-particle attractions to aggregate through van der Waal forces. Herein, the EE percentage of PZQ was observed to be 71.63% in the drug-loaded Compritol-lecithin-SLNs prepared using PF127 as a surfactant, and the LC percentage was 11.46%.

CLF and CLPF represent the formulations of Compritol-lecithin-solid lipid nanoparticles and PZQ-loaded Compritol-lecithin-solid lipid nanoparticles with PF127 as a surfactant.

PDI means polydispersity index, CLF and CLPF represent the formulations of Compritol-lecithin-SLN and PZQ-loaded Compritol-lecithin-SLN with PF127 as a surfactant. DEE percentage is the drug entrapment efficacy, and LC percentage is the loading capacity.

### 2.2. Evaluation of FTIR Spectroscopy

**Figure 2:**
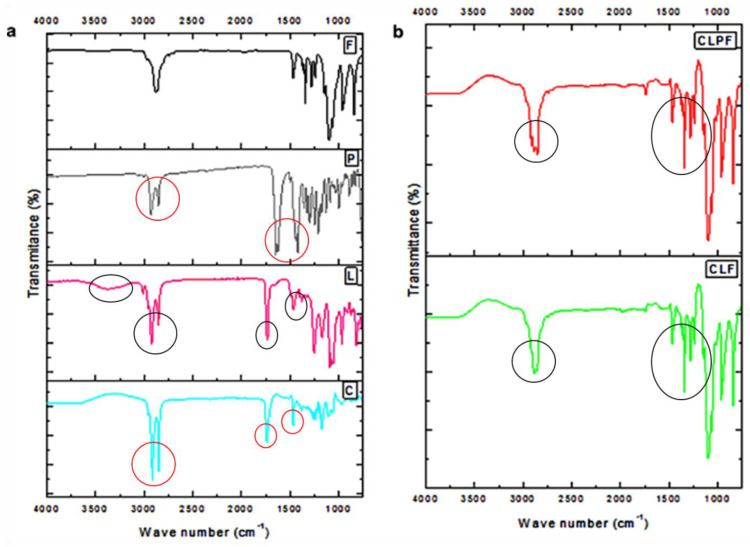
FTIR spectra of (**a**) all the excipients F-PF127, P-Praziquantel, L-Lecithin, C- Compritol (**b**) the formulated unloaded and PZQ-loaded SLNs.

The chemical characteristic of PZQ, stabilizer (PF127), lipids (Compritol 888 and lecithin), and the formulated SLNs were investigated by Fourier transform infrared (FTIR) spectroscopy. As shown in Figure 2a, Compritol 888 showed characteristic peaks at wavenumbers 2955.89 cm^−1^ to 2849.07 cm^−1^, 1736.42 cm^−1^, 1465.14 cm^−1^, and 719.26 cm^−1^ which correlated to O-H, -C-H(CH_2_), C=O, and -C-H(CH_2_) vibration, respectively. All the FTIR stretching frequencies correspond to pure Compritol 888, and the peaks are corroborated with other reported studies [19]. In lecithin, the peaks found in wavenumbers 3385.24 cm^−1^, 3011.09 to 2853.14 cm^−1^, 1734.19 cm^−1^, and 1466.14 cm^−1^ aligned with N-H, O-H, =-CH_3_ and C-H, C=O and P=O, respectively, and these peaks are similar to those obtained by Sumaila et al. [20]. Likewise, the vibration peaks found in PZQ at the wavenumbers 2929.49 cm^−1^ and 2852.52 cm^−1^ corresponded to the symmetric and asymmetric vibrations of CH and CH_3_, respectively, which are similar to the results of other reported literature [21,22].

When comparing native lipids with the Compritol-lecithin SLN (Figure 2b), a slight change was observed, and some peaks found in the native lecithin and Compritol were not found in the formulated SLNs. Other vibrational peaks found in SLNs, similar to those found in native lipids, may be due to electrostatic and hydrophobic interactions that occur between the two lipids and PF127 to form the SLN. Another possible interaction is hydrogen bonding, which occurs between the amine group and the bonds between the oxygen atom of the phosphate group of phospholipids, as well as hydrogen bonding between carbonyl oxygens. The slight change in the peaks found in wavenumbers 2887.88 cm^−1^, 2916.68 cm^−1^, and 2850.30 cm^−1^ of the SLNs may be a result of electrostatic repulsion, which in turn enhances the stability of the particles. Interestingly, a comparison of the peaks found in unloaded and drug-loaded SLNs showed that the characteristic peaks corresponding to PZQ were not found in the PZQ-loaded CLPF-SLNs. This means that PZQ is not found on the surfaces of SLNs. It is surmised that PZQ was molecularly dispersed, well encapsulated, and entrapped in the stable lipid matrix.

### 2.3. Evaluation of Mechanical Properties under Physiological Conditions

**Figure 3:**
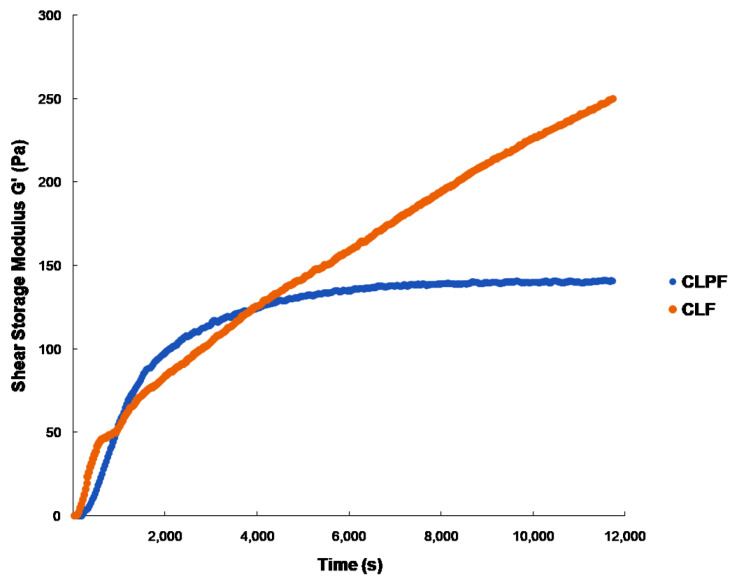
Kinetic gelation plots of unloaded and PZQ-loaded SLNs showing the shear storage modulus (G′) (Pa) observed over the periods of 3 h at 37 °C. CLF and CLPF represent the formulations of Compritol-lecithin-solid lipid nanoparticles and PZQ-loaded Compritol-lecithin-solid lipid nanoparticles with PF127 as a surfactant.

**Figure 4:**
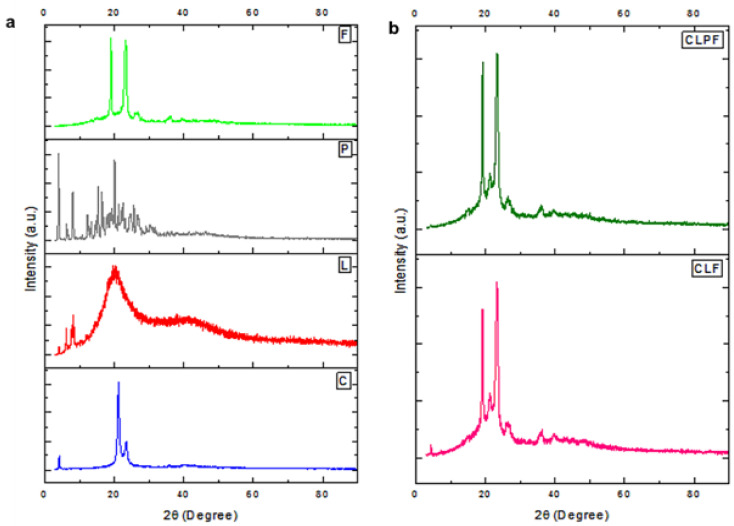
XRD diffractograms of (**a**) all the raw excipients F-PF127, P-Praziquantel, L-Lecithin, C-Compritol; and (**b**) the formulated unloaded and PZQ-loaded SLNs.

The viscoelastic properties of the prepared SLNs were evaluated using ElastoSens^TM^ Bio, which uses a non-destructive approach to characterize the evolution of the mechanical features of a formulation in real-time [17]. As shown in Figure 3, the gelation times for CLF and CLPF samples were 322.2 and 207.8 s respectively, after the measurements were taken every 30 s at 37 °C. The gelation times for all four formulations range between 2 min 4 s and 10 min 37 s. It was observed that the onset gelation time for SLNs loaded with PZQ had a relatively fast onset gelation time when compared with the SLNs unloaded with PZQ. As shown in Figure 4, the delay in the gelation time of the CLF-SLNs may be a result of several molecular interactions (electrostatic repulsion and hydrogen bonding) between the two lipids molecules to form a complex. It was shown that the addition of PZQ to produce CLPF increases the onset gelation in realtime in the SLNs loaded with PZQ; this may be a result of a quick complexation interaction between the lipids complex and the PZQ. More so, the result of that figure showed that the PZQ-loaded formulation has a rapid G′ and is gelled about an hour after the reaction. This may be attributed to the uninterrupted reaction between the molecules (amine and phosphate molecules) of the drug and lipids that facilitated the complexation process. At the end of the 2 h of viscoelastic measurements, CLF and CLPF attained G′ maximums of 286 Pa and 140 Pa, respectively. It was discovered that the drug-loaded formulation completed its gelation a few minutes before the 2 h reaction.

### 2.4. Evaluation of Crystal Nature of the Formulated SLNs

The crystal characteristics of the samples were evaluated using the XRPD technique. This analysis was carried out for all the excipients (the lipids, drug, and stabilizer) and the formulated SLNs for comparison. The patterns presented in Figure 4a,b depicted the patterns of all samples. It was shown that all the characteristic peaks of the excipient materials—that is, PZQ, Compritol, Lecithin, and PF127 in Figure 4a—corresponded to the reported peaks in the literature [19,20,21,23]. In Figure 4a, the major characteristic peaks at 2θ—19.5° and 23.8° of the stabilizer (PF127) displayed the highly crystalline nature of the stabilizer, and for the lipids, Compritol was shown to be highly crystalline with the diffraction peaks at 2θ—4.5°, 20.1°, and 23.2°. While lecithin depicted semi-crystalline, amorphous properties with the major characteristic peak at 2θ—20.4°, the broad amorphous peak at 2θ—40.8°, and other crystal peaks at 2θ—4.2°, 7.6°, and 10.3°, PZQ was shown to be highly crystalline in nature. As shown in Figure 4b, both the drug-loaded SLN and the bare SLN with PF127 (that is, CLF and CLPF) were shown to be highly crystalline in nature, which may be attributed to enough absorption of PF127 on the lipid matrix surface. Importantly, the corresponding diffraction peaks to pure PZQ were not found in both the PZQ-loaded SLNs with surfactant. This result further confirmed the encapsulation and the molecular presence of PZQ within the matrices of the lipid, correlated with FTIR and DSC.

### 2.5. Evaluation of Thermophysical Properties of the Formulated SLNs

**Figure 5:**
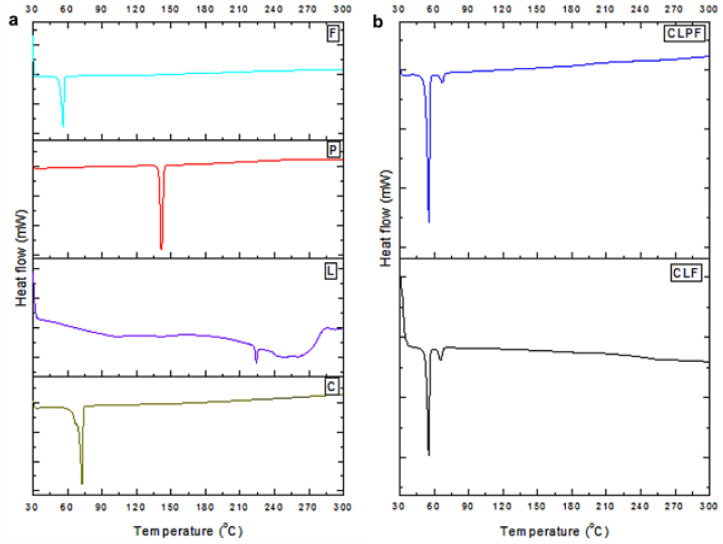
(**a**) DSC thermograms of all the raw excipients F-PF127, P-Praziquantel, L-Lecithin, C-Compritol; (**b**) the formulated unloaded and PZQ-loaded SLNs.

The thermal behaviour of the formulated SLNs was examined by DSC analysis, this was undertaken to ascertain any possible change in the physical features of the components of the individual SLNs’ formulation and to determine the amorphous or crystalline attribute of the PZQ-loaded SLNs. The data and resultant thermal bends are displayed in Table 2 and Figure 5, respectively. The melting points of the Compritol, lecithin, drug PZQ, and stabilizer PF127 were shown to have endothermic peaks at 71.83°, 223.08°, 137.99°, and 55.06°, respectively, which can be attributed to the fusion of their crystalline portions. All the raw excipients’ melting points were similar to the results documented in the literature [19,20,21,23]. The DSC thermograms also displayed the endothermic melting peaks of the formulated native SLNs and PZQ-loaded SLNs. For the SLNs made with PF127 as a stabilizer, minor endothermic melting peaks corresponding to the melting of the stabilizer were observed. The thermal response showed the complete melting of the lipid matrix and a slight shift in the melting of the stabilizer. In the PZQ-loaded SLNs (CLPF with melting peaks of 53.98), the endothermic melting peaks corresponding to the melting peaks of the native SLNs (CLF with the endothermic peaks of 53.53) were observed with the absence of the endothermic melting peaks corresponding to the drug; this is an indication that the drug PZQ is well properly dispersed or loaded within the core of lipid matrix.

### 2.6. Investigation of the Colloidal System of the Synthesized SLNs

**Figure 6:**
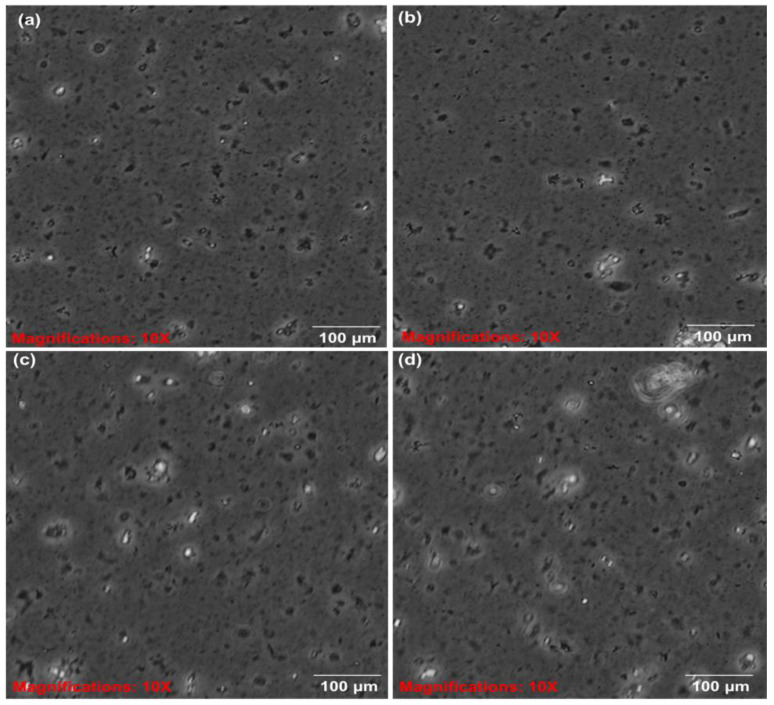
Inverted microscope images of colloidal aggregates of the (**a**,**b**) CLPF-SLNs and (**c**,**d**) CLF-SLNs (×10 magnification). CLF and CLPF represent the formulations of Compritol-lecithin-solid lipid nanoparticles and PZQ-loaded Compritol-lecithin-solid lipid nanoparticles with PF127 as a surfactant.

The colloidal system of the formulated SLNs was investigated using an inverted light microscope (Olympus CKX53, Tokyo, Japan) using the X objective lens. The microscope images showed that the particles are uniformly suspended with well-defined shapes and sizes, and form spherical surfaces as shown in Figure 6. Nanoparticles that are colloidal with size, composition, shapes, crystallinity, or surface properties and structure, and that are controllable, can be fine-tuned based on the applications they are needed for through their properties and characterizations [24,25]. The dispersion of the colloidal system could also contribute to the stability, interparticle interactions, microstructure, thermodynamic aspects, and transport properties of the nanoparticles. It has been reported that the dispersion of a colloidal system enhances the stability, thermodynamic properties, interparticle interactions, and microstructural aspect of particles, that is, if the dispersed particles remain as separate units and do not form a cluster or an aggregate [24,25].

### 2.7. Analysis of the In Vitro Release Behaviour of PZQ from the Formulated CLPF-SLN

**Figure 7:**
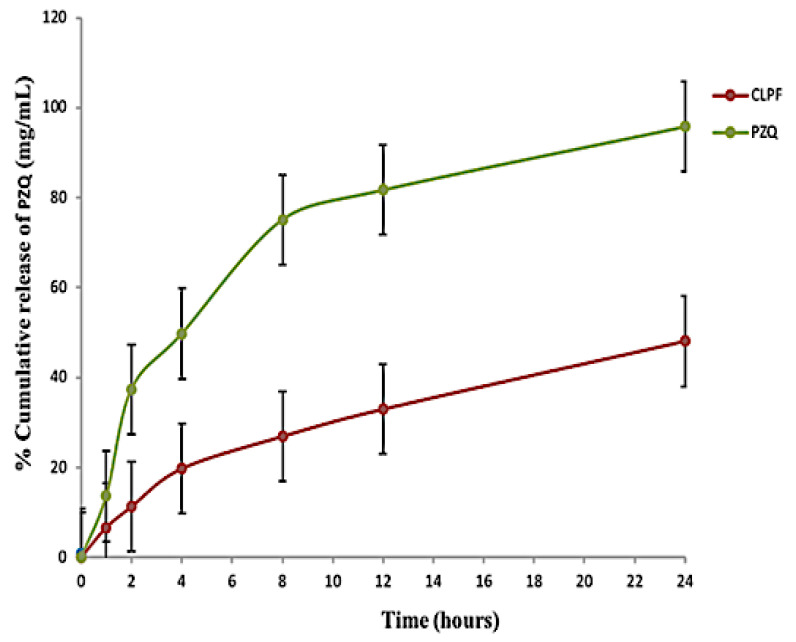
In vitro release profile of native PZQ, PZQ-loaded SLNs (CLPF). Temperature = 37 ± 1 °C, Release medium = PBS, pH = 7.4 (containing 0.002% Tween 80) (*n* = 3, mean ± SD).

**Figure 8:**
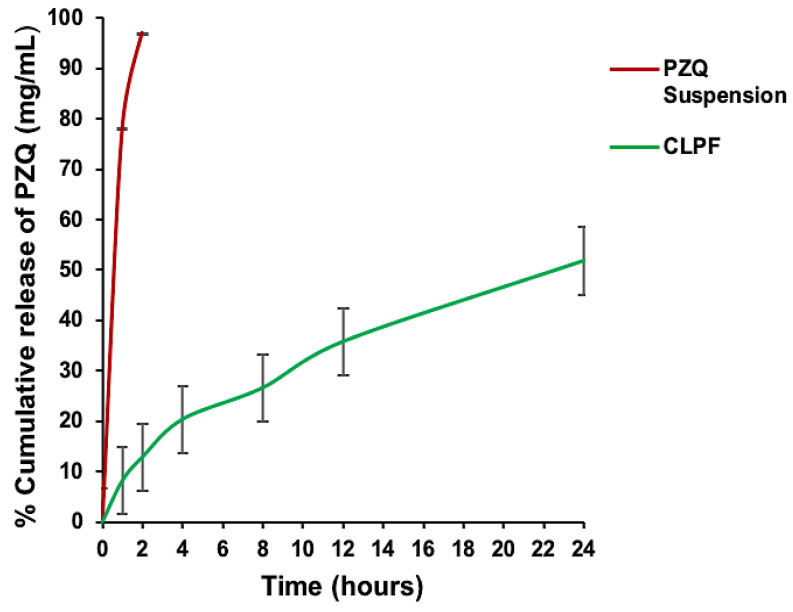
In vitro release profile of native PZQ suspension, PZQ-loaded SLNs (CLPF) after day 120. Temperature = 37 ± 1 °C, Release medium = PBS, pH = 7.4 (containing 0.002% Tween 80) (*n* = 3, mean ± SD).

The in vitro release behaviour of PZQ from the CLPF-SLNs formulated was examined together with the free PZQ. As shown in Figure 7, there was a burst release of PZQ in the initial stage (the first 2 h) of the free PZQ with about 37.3%, which was later followed by a steady release over the remaining 24 h with a total release of approximately 95.8%. This burst release could account for the rapid metabolism and quick conversion of the drug to its inactive form. There was a slow and steady exponential release of PZQ from the formulated SLNs over the 24 h within the pH medium tested. A cumulative release of 48.08% for CLPF was reached within 24 h at a physiological pH of 7.4. As shown in Figure 8, the in vitro release profile of PZQ within the CLPF suspension after a 120 day stability study was determined, and the PZQ release profile obtained was similar to that observed in the normal CLPF, but with a slightly higher cumulative release of about 51.8% within 24 h. Meanwhile, the in vitro release profile of PZQ suspension released almost all free PZQ within 2 h at 37 °C under sink conditions.

### 2.8. Particle Size Distribution, PDI, and Zeta Potential Analysis of the SLNs as a Function of Time

**Figure 9:**
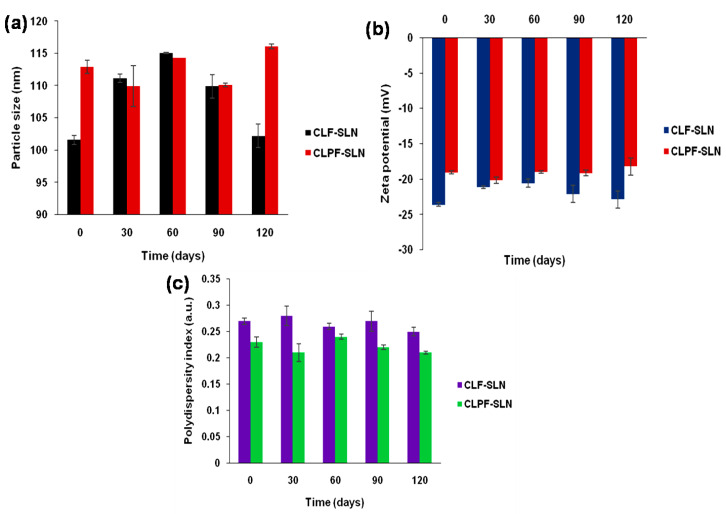
Characteristics of unloaded and PZQ-loaded CLF-SLN (**a**) Particle size, (**b**) zeta potential, (**c**) PDI at room storage temperatures over 120 days.

The physical stability of both the unloaded and the PZQ-loaded CLF-SLN was evaluated at room temperature over 120 days at 30 day intervals using dynamic light scattering (DLS) and electrophoretic light scattering (ELS), which measures the particle size, PDI, and zeta potential. Figure 9 shows the average particle size for CLF to be 101.60 ± 0.70, 111.13 ± 0.65, 115.03 ± 0.11, 109.90 ± 1.80, and 102.23 ± 1.81 nm for day zero, day 30, day 60, day 90, and day 120, respectively. The polydispersity index and zeta potential for CLF from day zero to day 120 ranges from 0.25 ± 0.008 to 0.28 ± 0.018 a.u. and −20.58 ± 0.56 to −23.60 ± 0.26 mV, respectively. The average particle size in the range of 109.96 ± 3.17–116.06 ± 0.37 nm (PdI = 0.21 ± 0.003–0.23 ± 0.010; zeta potential = −18.21 ± 1.22 to −20.16 ± 0.44 mV) was observed for the PZQ-loaded CLF-SLN from day zero to day 120.

### 2.9. Stability Analysis of Unloaded and PZQ-Loaded CLF-SLN through Turbiscan Technology as a Function of Time

**Figure 10:**
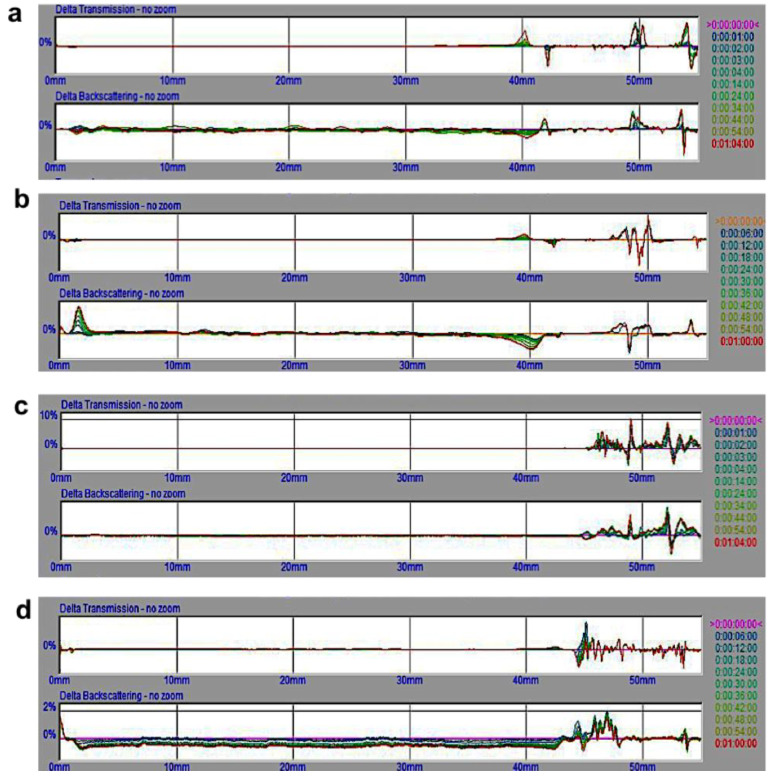
Presentation of the destabilization kinetics of (**a**) day 1 of CLF-SLN, (**b**) day 120 of CLF-SLN, (**c**) day 1 of CLPF-SLN and (**d**) day 120 of CLPF-SLN suspension, indicating ∆BS and T calculated at 25 ± 0.5 °C for 60 min duration.

The long-term stability of unloaded and PZQ-loaded CLF-SLN was investigated using a Turbiscan Lab Expert. The samples were scanned for 1 h at 6 min intervals for the dynamics of particle migration. As shown in Figure 10, the ΔT and ΔBS profiles of unloaded and PZQ-loaded CLF-SLN showed that there were no significant modifications in the ΔT and ΔBS signals for both samples during the first day and the last day throughout the length of the stability analysis, indicating a good stability profile. The backscattering signals noticed at the upper portions of the samples in Figure 10a–d were ignored since they also occurred at the same portions of the transmission signals, which suggests the impacts of secondary reflections on the glass.

### 2.10. Surface Morphology Characterization of the SLNs after 120 Days

**Figure 11:**
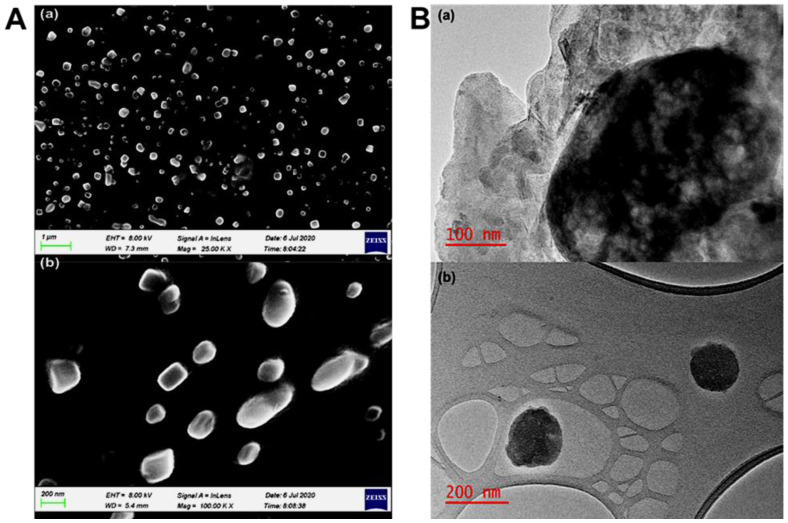
(**A**)SEM images of the (**a**) SLNs and (**b**) suspended drug molecules in the lipid matrices of the SLNs; (**B**) Transmission electron microscope (TEM) images of (**a**) SLNs at 100 nm (**b**) at 200 nm.

The morphological surface of the formulated SLNs was evaluated by SEM and TEM, and the SEM images showed the presence of discrete entities, along with the smooth, slightly spherical, and cube-like morphological surface of the SLNs (Figure 11A(a,b)). In Figure 11A(b), PZQ-loaded SLNs’ SEM images revealed the presence of a dark portion of the images of the matrix structure, which is an indication that the drug’s (PZQ) molecules are well dispersed within the matrices of the lipid, leading to the formulation of SLNs. A transmission electron microscope (TEM) was also employed to characterize the physical stability of the SLN particles after 120 days. The TEM images in Figure 11B showed that the particles are spherical in morphology and shape. Importantly, the morphology profile image for CLPF shown in Figure 11B(a) revealed that the drug’s(PZQ) molecules are still completely well dispersed within the lipid matrix of the nanoparticles, which could be presented as the chunky dark portion within the image of the matrix structure.

### 2.11. Analysis of In Vitro Cytotoxicity of SLNs

**Figure 12:**
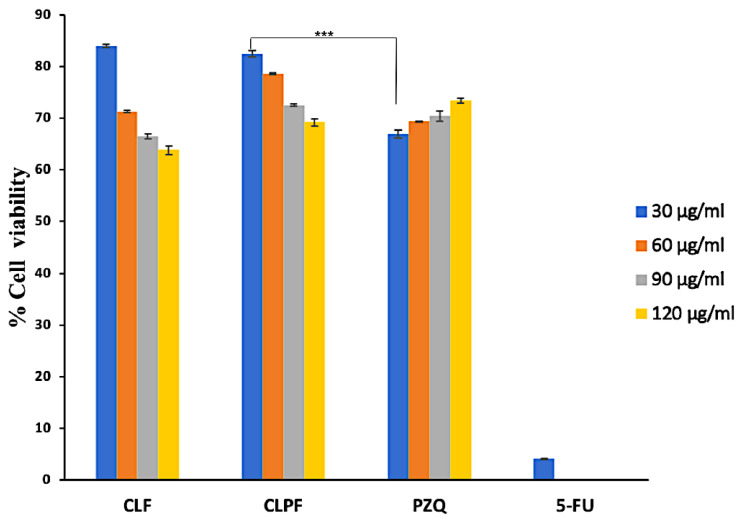
MTT assay measurement of the viability of RAW 264.7 murine macrophage cells after 72h treatment with free PZQ, with the formulated unloaded and PZQ-loaded SLNs at different concentrations (Data represent *n* = 3, mean ± SD; *** indicates *p*-value ≤0.0001 when compared to the same concentration of PZQ. CLF: Compritol-Lecithin-F127; CLPF: Compritol-Lecithin-PZQ-F127; PZQ: Praziquantel; 5-FU: 5-fluorouracil (10 µg/mL)).

The 3-[4,5-dimethylthiazol-2yl]-2,5-diphenyl tetrazolium bromide (MTT) examination was carried out to investigate the cytotoxicity of SLNs and PZQ on RAW 264.7 murine macrophage cells. This assessment was carried out by testing all the formulated SLNs and PZQ on the macrophage cell line for 72 h within 30 to 120 µg/mL concentration. The results showed that the formulated SLNs showed acceptable levels of cell viability, with no substantial cytotoxic effects on the RAW 264.7 cells. The highest concentration of CLF (120 µg/mL), which is way beyond what was observed in the drug release data, had about 66% viability, the lowest percentage of viability (Figure 12).

### 2.12. Analysis of RAW 264.7 Murine Macrophage Cells Morphology

**Figure 13:**
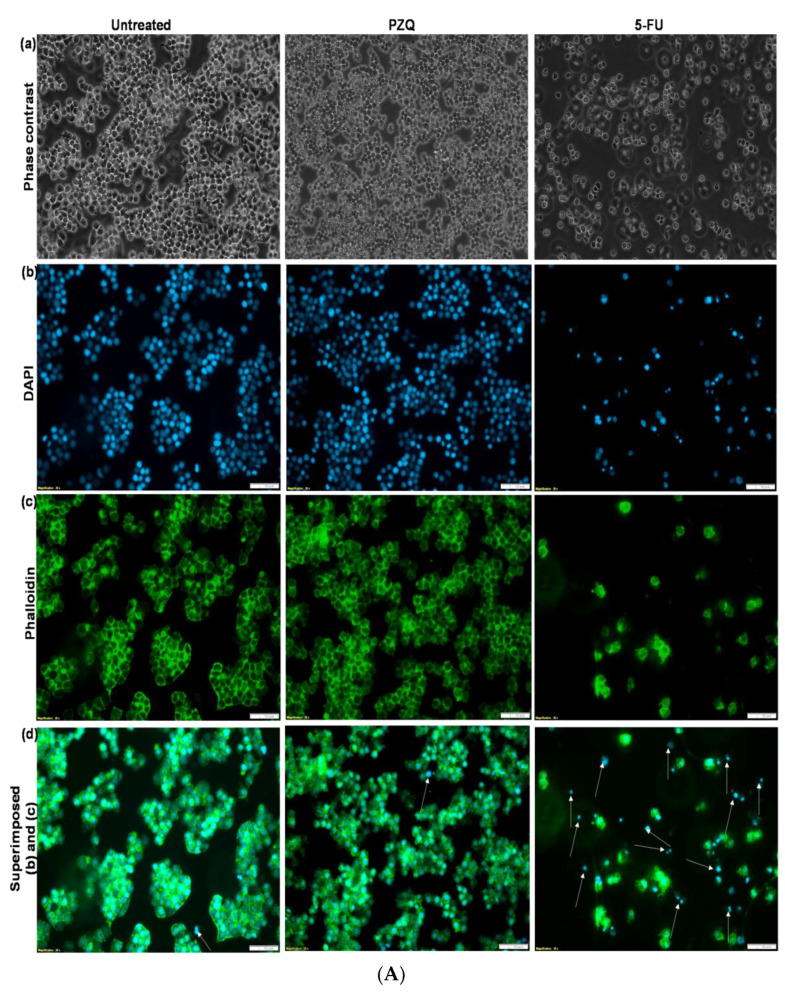

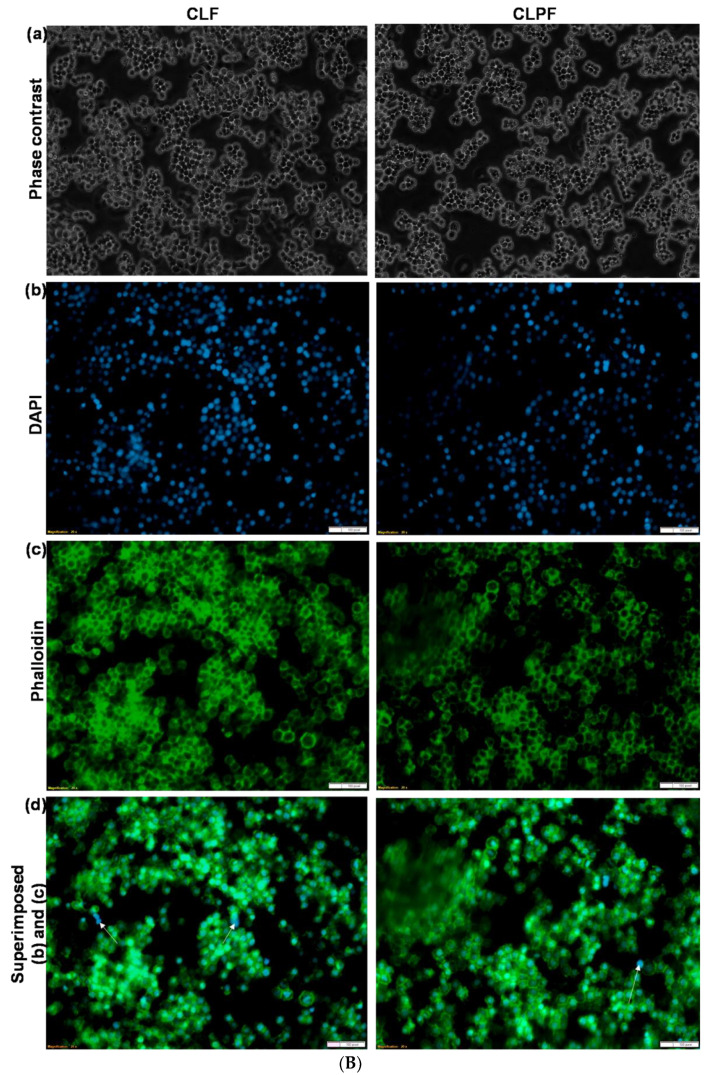
(**A**) Fluorescent microscopy images of RAW 264.7 macrophage cell morphology analysis (**a**) Phase contrast, (**b**) DAPI, (**c**) Phalloidin, and (**d**) Superimposed of (**b**,**c**) for untreated (control), cell treated with 90 µg/mL of PZQ and 5-FU (10 µg/mL) (negative control). Arrows denote dead cells. Scale bar: 100 µm; (×20 magnification). (**B**) Fluorescent microscopy images of RAW 264.7 macrophage cell morphology analysis: (**a**) Phase contrast, (**b**) DAPI, (**c**) Phalloidin, and (**d**) Superimposed of (**b**,**c**) with 90 µg/mL of CLF and CLPF. Arrows denote dead cells. Scale bar: 100 µm; (×20 magnification).

Figure 13a,b presented the cell morphology of the RAW 264.7 macrophage cells after being treated with 90 µg/mL of the prepared SLNs, PZQ, and 10 µg/mL of 5-fluorouracil (5-FU) as the positive control. The cells were visualized under an inverted microscope (Olympus CKX53) for phase-contrast images and a compound fluorescent microscope (Olympus IX51) for fluorescence images. It was revealed by the phase-contrast images that all the treated cells showed normal RAW 264.7 cell morphology with a round and smooth shape, whereas the cells treated with 5-FU as the negative control form pseudopodia, which resulted in cell death. It was further shown by the fluorescence microscopy analysis of the DAPI and phalloidin images that the prepared SLNs pose no cytotoxicity effect on the cellular morphology of RAW 264.7, because the DAPI revealed the nucleus of the cells, while the phalloidin showed the cytoplasm membrane of the cells. Additionally, the overlaid DAPI and phalloidin images showed that the nuclei are well positioned within the cytoplasm of the untreated PZQ and SLNs cells. Ruptures in the cytoplasm membrane and the exposure of the nucleus were observed in those treated with 10 µg/mL of 5-FU as the negative control.

### 2.13. In Vivo Toxicity Evaluation

**Figure 14:**
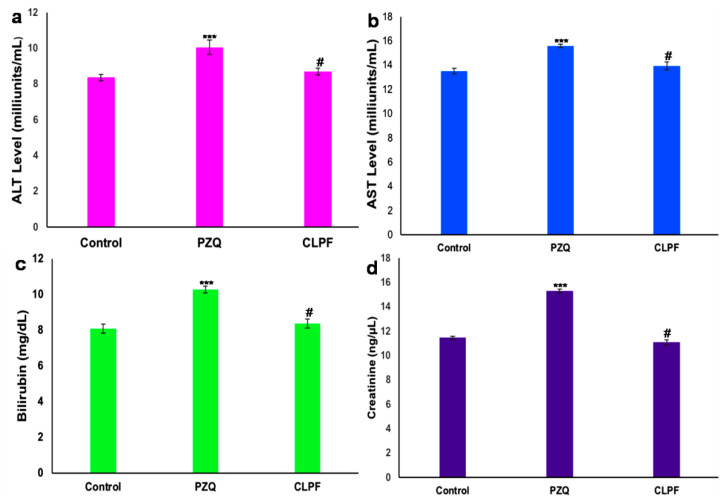
Biochemical markers (**a**) ALT (**b**) AST (**c**) Bilirubin and (**d**) creatinine levels in plasma. Values are expressed as mean ± standard deviation of five determinations (Data represent *n* = 3, mean ± SD). *** indicates *p* < 0.0001 when compared to the control group, and # indicates *p* < 0.0001 when compared to the PZQ group.

The extent of the liver and kidney damage of PZQ-loaded CLF-SLN and native PZQ has been evaluated through the analysis of the different biochemical markers [26] (ALT, AST, bilirubin, and creatinine) levels in the blood plasma in the rat model. As shown in Figure 14, the ALT levels in the control group, PZQ group, and CLPF group were observed to be 8.37, 10.05, and 8.7 milliunits/mL, respectively; 13.51, 15,70, and 13.93 7 milliunits/mL were observed for the AST levels in the control, PZQ, and CLPF groups, respectively. More so, both the bilirubin and creatinine levels were observed to be 8.09, 10.28, and 8.37 mg/dL, and 11.47, 15.32, and 11.09 ng/µL for the control groups, PZQ groups, and CLPF groups, respectively. From the results, a significant increase (*p* < 0.0001) in the levels of biochemical markers was observed in the plasma of the groups of rats that received PZQ as compared to the control. In addition, the plasma of the CLPF-receiving group showed a significant decrease (*p* < 0.0001) in the levels of all the biochemical markers when compared to the PZQ group. This showed that the PZQ-loaded CLF-SLNs do not cause any oxidative stress damage to the rats [26,27].

### 2.14. Histopathological Analysis

**Figure 15:**
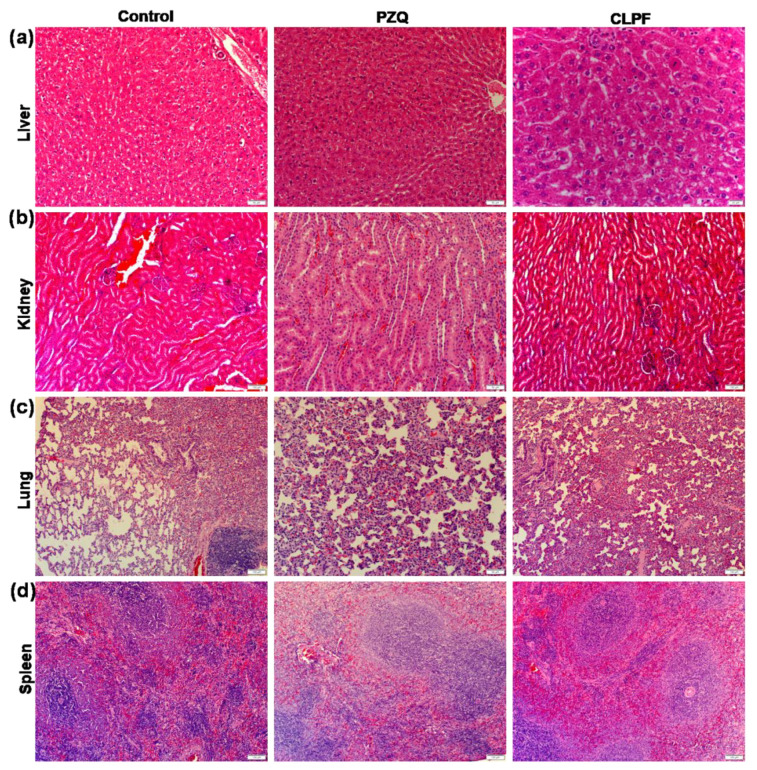
Histopathological images of (**a**) liver, (**b**) kidney, (**c**) lung, and (**d**) spleen after treating uninfected rats with 250 mg/kg of PZQ and CLPF (×20 magnification). CLPF: PZQ-loaded Compritol-lecithin-solid lipid nanoparticles with PF127; PZQ: Praziquantel.

As shown in Figure 15, the liver of an untreated control group showed moderate granularity in the hepatocyte cytoplasm, and the PZQ group depicts marked granularity in the hepatocyte cytoplasm. Hepatocytes show marked granularity of the cytoplasm in the CLPF group, with a few periportal hepatocytes in a few areas which appear mildly vacuolated with minimal swelling and a single blood vessel containing fibrin in the lumen. In the histopathological analysis of the kidneys, the untreated control group showed a normal histological appearance of the kidneys, while a few deep cortical tubular epithelial cells with a slightly granular cytoplasm were observed in the PZQ group; meanwhile, the kidneys in the CLPF group appear histologically normal.

The lung histological examination of an untreated control group depicts multifocal extensive alveolar atelectasis, and alveolar capillaries show multifocal moderate leukostasis of mononuclear cells and the presence of few multifocal foamy macrophages in the alveolar walls. Additionally, few bronchial-associated lymphoid tissue follicles (BALT) are visible and appear expanded. Moderate to severe diffuse alveolar atelectasis and alveolar capillaries were observed in the PZQ group, and show mild mononuclear leukostasis, with some active presence of BALT follicles. The CLPF group followed a similar pattern with a moderate diffuse alveolar atelectasis and the appearance of mildly active BALT. These lung histopathological results correlated with the experimental design and procedure because blood was collected from the heart, which may cause pulmonary atelectasis, and edema could also develop terminally following euthanasia. Normal active lymphoid tissue was shown in the spleen of all the groups, and the abnormalities are not visible.

### 2.15. Evaluation of Parasitological Cure Rate

**Figure 16:**
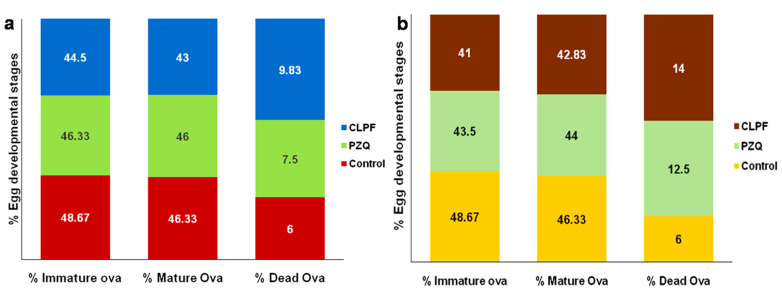
Effects of PZQ and CLPF:(**a**) (single dose 250 mg/kg two weeks post-infection) on % egg developmental stages in *S. mansoni*-infected mice euthanized six weeks post-infection; (**b**) (single dose 250 mg/kg four weeks post-infection) on % egg developmental stages in *S. mansoni*-infected mice euthanizedsix weeks post-infection.

Table 3 and Table 4, and Figure 16a show the data for worm recovery, oogram pattern, and egg count in tissues for two weeks post-infection treatment. As shown in Table 3, a statistically significant difference in the percentage reduction in the total worm burden was observed in the liver and portomesenteric vein following the oral administration of 250 mg/kg single dose of CLPF compared to the PZQ-treated group. The oral administration of CLPF (250 mg/kg single dose) showed a statistically significant reduction (*p* < 0.0001) in the percentage of mature and immature ova, as well as a percentage increase in the dead ova (Figure 16b) when compared to the infected untreated control and PZQ groups. The percentage reduction in the ova count in the liver and intestine depicted in Table 4 showed a statistically significant difference (*p* < 0.0001) in the CLPF-treated group when compared to the PZQ and the infected untreated control groups two weeks post-infection.

The data for worm recovery, egg count in tissues, and oogram pattern for four weeks post-infection treatment are shown in Table 5 and Table 6, and Figure 16b. As demonstrated in Table 5, the CLPF group had a statistically significant reduction in the percentage of total worm load in the liver and portomesenteric compared to the PZQ-treated group. When compared with the infected untreated control and PZQ groups, a single dosage of CLPF of 250 mg/kg resulted in a statistically significant increase (*p* < 0.0001) in the percentage of dead ova and a decrease in mature and immature ova in *S. mansoni*-infected mice (Figure 16b. Table 6 shows that when a single dosage of 250 mg/kg CLPF was given two weeks after infection, the percentage reduction in ova count in the liver and intestine was statistically significant (*p* < 0.0001) when compared to the PZQ and the infected untreated control groups.

## 3. Discussion

Over the years, PZQ has been the main drug utilized for schistosomiasis treatment, and the drug has been effective and well tolerated by the affected populations because of its ability to reduce the parasitic load of all species causing schistosomiasis. Additionally, PZQ reduces several symptoms of Schistosoma infection, and it is effective against adult schistosome worms [2,28].

PZQ has shortcomings, which include the inability to kill the juvenile schistosomes and a rapid metabolism that lowers the bioavailability of the drug’s molecules in systemic circulation. Another concern is the development and increase in drug resistance, particularly since decreased susceptibility has frequently occurred both in the laboratory and in the field [2]. Thus, there is an urgent need to improve some properties of PZQ, such as increasing its bioavailability and improving its release rate using the drug delivery systems approach. These improvements could fight parasite resistance, increase the uptake of the drug into the bloodstream following ingestion and circumvent vast first-pass metabolism by extending the release rate of the drug over time.

SLNs synthesized from one and/or two or more blends of solid lipids have been approved as drug carriers for over two decades [10,11]. This type of drug nanocarrier is more advantageous than others, such as polymeric nanoparticles, liposomes, and emulsions, because the matrices of the solid lipid can protect the chemical instability of the drugs. SLNs offer the controlled release of drug patterns when likened to nanoemulsions; they also present nanosuspension which is more stable than liposomes. Additionally, the wide application of SLNs in oral, dermal, intravenous, and pulmonary [11,12] areas when compared to polymeric nanoparticles is because they are made of excipients that are generally recognized as safe and physiologically well tolerated with reduced cytotoxicity.

Thus, the formulation of SLNs using Compritol and lecithin was demonstrated in this study through solvent injection co-homogenization techniques for the delivery of PZQ to enhance the therapeutic effectiveness of PZQ in the treatment of schistosomiasis. The temporal stability was also studied. In this study, the particle size for the formulations is uniform and falls within the size range of a suitable nano-delivery system [9,10,11]. The particles for a nanocarrier must be uniform; for many colloidal drug delivery systems designed from solid lipids, the sizes range between 50 and 1000 nm [29]. The polydispersity index of the formulations showed the phospholipid vesicles’ homogenous population of the SLN. In the use of lipid-based carriers in the applications of drug delivery, a PdI equal to 0.3 or less is believed to be satisfactory and depicts phospholipid vesicles with a homogenous population [30]. The zeta potential data showed that the formulations are stable regarding aggregation, flocculation, sedimentation, coagulation, and creaming via a strong electrostatic repulsion that keeps the particles charged from one another. The percentage of EE of PZQ was observed to be 71.63% in the drug-loaded Compritol-lecithin-SLNs prepared using PF127 as a surfactant and the percentage of LC was 11.46%. This result corroborated all other physicochemical characterization results which showed that PZQ is not found on the surface of the SLNs and that PZQ molecules were well dispersed and encapsulated in the stable matrices of the lipids.

In this study, the slower or sustained PZQ release from SLNs could be attributed to the sustained-release property of SLNs, and this could further buttress other physicochemical results that show PZQ is well dispersed and encapsulated within the lipid matrix. Thus, this slower and sustained release could be of importance in enhancing the therapeutic effect of the drug, since it has been stated that the rapid metabolism and quick conversion of the drug to its inactive form could be the major reason why the drug fails to kill the juvenile worms [3,31]. This sustained-release can also provide extended-release kinetics, which can lead to the reduction in the frequent administration of the drug, which will, in turn, enhance patient compliance, most especially when treating infectious disease conditions [20], or in this case, schistosomiasis.

Drug stability has an impact on the efficacy and safety of the drug substance; degradation impurities could cause efficacy loss and potentially harmful consequences. As a result, attaining the physical and chemical stability of drugs is critical to ensuring their safety and quality [13]. DLS and ELS measurements did not show any significant influence on the average particle size, PdI, and zeta potential of both the unloaded and PZQ-loaded CLF-SLN from day zero to day 120 of the stability analysis at room temperature, which was also revealed in the surface morphology and in vitro drug release analysis. This indicates that the addition of PF127 as the surfactants provided long-term stability for Compritol ATO 888-lecithin solid lipid nanoparticles nanosuspension over 120 days at room temperature, and this corroborated with other studies that PF127 promotes and enhances the stability of nanoparticles [14,15,16]. Furthermore, turbiscan data corroborated the dynamic light scattering analysis, which showed no significance in the variation in the particle sizes of the sample solutions.

Interestingly, it can be further averred that the decrease in percentage viability of cells treated with the formulated SLNs is dose-dependent. Although RAW 264.7 cells showed acceptable cell viability levels following PZQ treatment, there was significantly lower cell viability (*p-*value < 0.0001) following treatment with 30 µg/mL CLPF when compared with that of 30 µg/mL PZQ. In other words, even though a dose-dependent decrease in cell viability was seen in SLNs, the opposite effect was observed in PZQ: an increase in cell viability with increasing concentration. Hence, the data suggest that macrophages may proliferate with increasing concentration of PZQ; an observation that was not made in formulated SLNs. In a similar vein, one research study indicated that concentrations less than 80 µM of the R enantiomer of PZQ could promote the proliferation of RAW 267.4 macrophages [32]. Thus, this dose-dependent increase in macrophage viability following PZQ treatment calls for further investigation, given that the mechanisms by which PZQ exhibits its antiparasitic effects and drug resistance are not fully understood.

The findings from the in vivo toxicity examination corroborated the results obtained by the in vitro cell viability study, which showed by histopathological and biochemical marker analyses that there is no toxicity or lesser oxidative stress, with the formulated CLPF having the ability to confer hepatoprotective effects on the animals. The histopathological and biochemical marker analysis data further suggested CLPF-SLNs possess the ability to circumvent traces of toxicity associated with bioactive molecules, because studies have reported that the excipients that were used were not to toxic [33,34,35]. In a parasitological study, the cure rate of CLPF and PZQ was determined, and it was revealed that treatment with 250 mg/kg CLPF had a stronger action on total worm load and ova count in both the intestine and the liver. In addition, when compared to the same dose of PZQ, the CLPF nanosystem had a greater effect on both mature and immature ova, and caused ova death during the egg developmental stages in both young and adult worms, represented by two and four weeks post-infected mice, respectively. The higher rate and increased absorption of the CLPF nanosystem by the worms and eggs in the liver, porto-mesenteric, and intestine may be responsible for the huge impacts of the CLPF nanosystem on schistosome eggs and worms. A similar observation was reported in an indirect membrane immunofluorescence study (IF), which involved the incubation of *S. mansoni* lung-stage larvae in 90% corn oil for 6 h, and the veiled apical membrane antigen interaction with anti-schistosome antibodies [36].

## 4. Materials and Methods

### 4.1. Material and Reagents

Glyceryl behenate (Compritol 888 ATO) was procured as a free sample from Gattefosse SAS (Saint-Priest, CEDEX, France). Praziquantel was purchased from LeapChem Co. Ltd.(Hong Kong, China), L-α-lecithin soybean which is composed of less than 2% triglycerides and 94% phosphatidylcholine). PF127, phosphate-buffered, 49409 Atto 488 Phalloidin, was bought from Sigma-Aldrich (St. Louis, MO, USA). RAW 264.7 cell line was procured from ATCC (Manassas, VA, USA), and ethanol and chloroform were obtained from Merck Pty Ltd. (Modderfontein, South Africa). The DAPI stain were purchased from Thermofisher (Waltham, MA, USA). All other components and chemicals utilized in this study were of analytical grade. For all the preparations in the study, double deionized water (DDW) was employed. Biochemical assays (AST, ALT, ALP, bilirubin, and creatinine) kits were purchased from Sigma-Aldrich (St. Louis, MO, USA).

### 4.2. Preparation of Compritol-Lecithin SLN-Loaded Praziquantel

**Figure 17:**
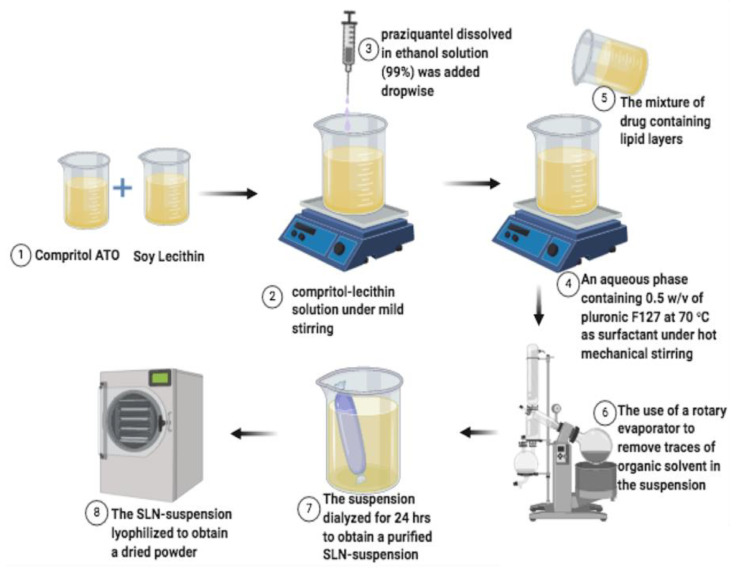
The schematic overview showing the preparation procedure for praziquantel-loaded Compritol-lecithin SLNs.

SLNs were prepared by employing solvent injection co-homogenization techniques described, as follows. Briefly, a Compritol-lecithin solution was prepared by dissolving the Compritol and lecithin in a ratio of 1:1 (100 mg) in 20 mL of chloroform. Thereafter, in 10 mL of ethanol solution (99%), 40 mg of praziquantel was dissolved and added dropwise to Compritol-lecithin solution under moderate stirring conditions. The mixture of drug-containing lipid layers was then injected into the aqueous phase containing 0.5 *w*/*v* of PF127 at 70 °C as surfactant under hot mechanical stirring to properly homogenize the suspension. Subsequently, the traces of organic solvent in the suspension were completely evaporated by using a rotary evaporator (Rotavapor^®^ RII, BüchiLabortechnik AG, Flawil, Switzerland) at 70 °C. The resultant SLN suspension was dialyzed overnight to purify the generated products. In a similar manner, PZQ unloaded-Compritol-lecithin emulsion was made following the same method and served as the control. The schematic diagram showing the preparation procedure is shown in Figure 17. The suspension was recovered and kept for further analysis at room temperature.

### 4.3. Determination of Particle Size Distribution, Polydispersity Index (PDI), and Zeta Potential

The SLN suspension kept at room temperature was used in ascertaining the zeta potential, average particle size, and polydispersity index (PDI) of the SLNs using Malvern ZetaSizer Nano ZS (Malvern Instruments, Worcestershire, UK) over 120 days. This was achieved by preparing samples of 0.1–0.2 mg/mL of final concentration from the SLN suspension using distilled and filtered water (0.22 µm), and about 2 mL of the sample of SLNs prepared was placed in cuvettes and measured. All measurements were carried out in triplicate.

### 4.4. Evaluation of the Drug Entrapment Efficacy and Drug Loading Capacity

The entrapment efficiency of PZQ trapped within the Compritol-lecithin SLN was evaluated. Briefly, the lyophilized SLN sample was dissolved and ruptured in an adequate amount of chloroform. Thereafter, the concentration of the PZQ was calculated at a wavelength of λmax = 265 nm using ultraviolet-visible spectroscopy (UV-Vis Spec) (Specord40, Analytik Jena, AG, Jena, Germany) and computed using a standard linear curve of PZQ in concentrations between 0 and 16 µg/mL in double-distilled water (R^2^ = 0.99). The percentage values of DEE (drug entrapment efficacy) and loading capacity (%LC) of the SLNs were carried out in triplicate and computed using these Equations below:

(1) $$\%\mathrm{DEE}=\frac{A_{q}}{T_{q}}\times 100$$

where $A_{q}$ represents the actual amount of PZQ measured by UV spectrophotometry and $T_{q}$ denotes the theoretical amount of PZQ mixed with the Compritol-lecithin SLN formulation.

(2) $$\%\mathrm{LC}=\frac{A_{q}}{W}\times 100$$

where $A_{q}$ represents the actual amount of PZQ measured by UV spectrophotometry and W denoted the weight of Compritol-lecithin SLN.

### 4.5. Evaluation of Fourier Transform Infrared Spectroscopy (FTIR)

Native PZQ, lecithin, Compritol, Compritol-lecithin SLN-loaded and unloaded PZQ, and Compritol-lecithin SLN-loaded PZQ with surfactant in powdered form were analysed to determine possible structural interactions between the lipids and the PZQ. The formulations were analysed through the use of Spectrum 2000 ATR-FTIR (PerkinElmer 100, Llantrisant, Wales, UK) with the wavelength ranging between 4000 and 650 cm^−1^ and with a 4 cm^−1^ resolution.

### 4.6. Mechanical Properties Analysis

The mechanical characteristics of the different SLN formulations were investigated under physiological conditions using the non-destructive vibration of acoustic and laser determination of responses from rheology without the sample contact method previously described by [37]. A total of 10 mg of the dried SLN was briefly dissolved in 5 mL of PBS (pH 7.4); thereafter, 3 mL of the suspension were loaded into the detachable sample holder designed specifically for ElastoSens^TM^ Bio2 (Rheolution, Montreal, QC, Canada). The real-time storage modulus for the complexation reaction of the suspension in the sample holder was performed for 2 h at 37 °C using ElastoSens^TM^ Bio2. The device was used for each sample, every 30 sand the storage modulus was determined. The procedures for each sample at physiological conditions were performed in triplicates.

### 4.7. X-ray Powder Diffraction (XRPD) Evaluation

The degree of crystallinity natures of the native PZQ, lecithin, Compritol, PF127, Compritol-lecithin SLN loaded and unloaded PZQ, and Compritol-lecithin SLN loaded PZQ with surfactant in powdered form were evaluated by XRPD spectra (Rigaku Mini Flex 600, Tokyo, Japan) fortified with CuKα radiation at 15 mA and 40 kV. The 2θ scan ranged from 3° to 90° was selected at a scanning rate of 10° per minute and was used to obtain diffractograms of the formulations.

### 4.8. Analysis of Differential Scanning Calorimetry (DSC)

Thermal behaviour of native PZQ, PF127, lecithin, Compritol, Compritol-lecithin SLN loaded and unloaded PZQ, and Compritol-lecithin SLN loaded PZQ with surfactant in powdered form were analysed on a DSC by Mettler Stare system provided with STARe SW software (Mettler-Toledo International Inc. 1900 Polaris Parkway Columbus, OH, USA). This task was achieved by weighing 3 to 6 mg depending on each sample in an alumina crucible of a pinhole and heating from 30 to 300 °C with a heating rate of 5 °C/min under a nitrogen flow.

### 4.9. Colloidal System Analysis of the Formulated SLNs

The colloidal system of the SLN dispersions was analysed under an inverted compound light microscope (Olympus CKX53, Olympus Corporation, Tokyo, Japan). The dispersion of the SLNs increased, and the surface and the attractive component of the interparticle interaction of the formulated SLNs were observable. This colloidal dispersion was determined to ascertain how the SLNs can be prevented from aggregation or flocculation.

### 4.10. In Vitro Analysis of PZQ Release from the Formulated SLNs

To evaluate the in vitro release behaviour of PZQ in free PZQ (thirty milligrams), PZQ-loaded SLNs were weighed and dispensed into specimen vessels containing 100 mL of pH 7.4 PBS (containing 0.002% Tween 80) and incubated at 37 ± 1 °C in a horizontal orbital shaking incubator (type LM-530, Yihder Technology Co., Ltd., New Taipei City, Taiwan), set at 25 rpm for 24 h. A minute before sampling, agitation was stopped to allow the SLNs to settle and to reduce the number of intact particles removed from the dissolution medium. Sampling was conducted at stipulated periods (1, 2, 4, 8, 12, and 24 h) and all aliquots withdrawn were centrifuged and diluted appropriately before UV-Vis spectrophotometric analysis at 265 nm. The experiments were conducted in triplicate on all the formulated PZQ-loaded SLN and free PZQ.

To determine the in vitro release behaviour of PZQ from CLPF suspension after day 120 of the stability study, a similar method described above was chosen, usinga dialysis technique and 20 mg/mL of PZQ suspension as a control. The in vitro release analyses of the PZQ were conducted in triplicate.

### 4.11. Optical Characterization and Stability Profiling of the Suspensions of SLNs Formulation

The stability and optical interactions of the SLN suspension on the first and last day of stability analysis were evaluated using the TurbiscanTM LAB (Formulaction, L’Union, France). Briefly, about 20 mL of CLF-SLN and CLPF-SLN suspensions were aliquot and introduced into specified sample holders and evaluated at fixed intervals (6 min) over 1 h duration at 25 ± 0.5 °C. The instrument was equipped with two detectors, the backscattering and transmission detectors, which are positioned at 54° and 180°, respectively. Moreover, the device was fixed with a pulsed near infra-redlight source, which travels along the sample collecting data vertically at 40 μm-intervals. The transmission detector revealed the light that was transmitted via the suspended SLN formulation, while the backscattering detector exposed the light that was recovered/rebounded by the suspended SLN formulation. The delineated SLN formulation stability was obtained from Turbiscan^TM^ LAB through the measurements of the changes in backscatter (∆BS).

### 4.12. Scanning Electron Microscopy and Transmission Electron Microscopy and

The morphological examination of the formulated SLNs was analysed by scanning electron microscopy (SEM). The lyophilized SLN samples were dissolved in distilled water and a drop was positioned on an aluminum specimen stub and left overnight to dry. Subsequently, the samples loaded on the stub were sputter-coated with both gold and palladium and shielded with a double-coated carbon glue disc for 4 min at 20 KV. The SEM pictures of the formulated unloaded and PZQ-loaded SLNs were generated with the help of SEM (Zeiss Electron Microscopy, SIGMA VP, Carl Zeiss Microscopy Ltd.; Cambridge, UK).

High-Resolution Transmission Electron Microscopy (HRTEM) version TECNAIF3OST-TEM was employed in ascertaining the morphology of the CLPF after 120 days. The CLPF suspension was concentrated to about 1:10 with distilled water and filtered using 0.22 µm. A drop of the concentrated CLPF suspension was mounted on the carbon-coated copper grid for 5 min followed by the excess removal of liquid, which was attained by blotting and thereafter air-dried at room temperature. Subsequently, the films on the copper grid were visualized under an electron microscope at 10,000× magnification.

### 4.13. In Vitro Cytotoxicity Assay (MTT Assay)

The in vitro cytotoxicity of the SLNs on RAW 264.7 macrophages was investigated through an MTT cell viability study. DMEM supplemented with 1% penicillin-streptomycin and 10% fetal bovine serum was utilized to culture the cells at 37 °C and 5% CO_2_ (in a humidified incubator). After every two days, the spent growth medium was replenished with a fresh growth medium until the cells reached about 80 to 90% confluence. The cells at a density of 1 × 10^5^ cells per well were seeded in 96well plates. After 24 h, the adhered cells were treated with SLNs and PZQ of 30–120 µg/mL concentration range in triplicates. Subsequently, the cells were incubated for 72 h in a humidified incubator at 37 °C and 5% CO_2_. After 72 h, 10 µL of MTT reagent (Merck, Darmstadt, Germany) was included in the wells and the cells were further incubated for 4 h at 37 °C. Thereafter, 110 µL of DMSO was added to the well plates and incubated subsequently at 37 °C for an hour so as dissolve the formed crystals formazan. Thereafter, the absorbance at 570 nm with a reference wavelength of 690 nm was measured by a multimode microplate reader (Victor X3 Multimode Multilabel Microplate Reader 2030-0030, Perkin Elmer, Waltham, MA, USA). The cell viability percentage was determined from the ratio of absorbance of the test samples to that of the untreated sample.

### 4.14. Evaluation of Cell Morphology

After RAW 264.7 murine macrophage cells had reached 80 to 90% confluence, 2.5 × 10^6^ cells/mL were seeded and cultured on coverslips in 6well plates containing 2 mL DMEM supplemented with 1.0% (*v*/*v*) penicillin-streptomycin antibiotic and 10% (*v*/*v*) FBS, and incubated for 24 h at 37 °C under 5% CO_2_. After 24 h, the adherent cells were treated in triplicates with 90 µg/mL of CLF, CLPF, and PZQ, as well as 10 µg/mL of 5-fluorouracil (5-FU) as a negative control. The cells were then cultured for another 24 h in a humidified incubator at 37°C and 5% CO_2_. The phase contrast morphology of the cells was observed under an inverted compound light microscope after 24 h of treatment (Olympus CKX53, Olympus Corporation, Tokyo, Japan). After that, the cells were fixed; this was achieved by first spiking the media growth containing the cells with 500 µL of 4% formaldehyde to avoid harm from the rapid shift in osmolarities between the fixation solution and the culture medium. After about 2 min, the medium was aspirated and decanted, and the cells were then fixed with pure 1 mL of 4% formaldehyde for about 20 min. After 20 min of fixation, the fixed cells were gently washed four times with 2 mL PBS to remove any unbound fixation agent, then stained with 500µL of a 50 µM fluorescent phalloidin solution to bind the F-actin cytoskeleton of the cell membrane for about 40 min at room temperature in the dark. After 40 min, the cells were washed four times with PBS to remove any unbound phalloidin stain, then stained with 500 µL of 500 nM DAPI stain solution to bind the nucleus of the cells and incubated at room temperature for 5 min in the absence of light. After that, the cells were carefully washed with PBS to eliminate any remaining DAPI stain solution. The coverslips were then placed on glass slides and examined using a compound fluorescence (×20 magnification) microscope (Olympus IX51, Olympus Corporation, Tokyo, Japan).

### 4.15. In Vivo Toxicity Evaluation

The evaluation of in vivo toxicity was performed in Sprague Dawley rats with a weight range of 250–300 g, kept in standard husbandry and housing conditions at normal room temperature, and provided with normal tap water and normal rat chow. The animals were assigned to three groups randomly, and each group was named as follows: Group I: control, Group II: PZQ, and Group III: CLPF. An equivalent amount of the dose (250 mg/kg) in about 2 to 3 mL of PZQ and CLPF was administered to the animals via a once-off oral gavage orally. Seven days after, all the animals were humanely decapitated, and their blood was collected via cardiac puncture, stored in microcentrifuge heparin tubes, and centrifuged for 5 min at 2500 rpm at 4 °C to isolate plasma from red blood cells (RBCs). Thereafter, the plasma was examined for a liver functioning test to assess the hepatotoxicity of the various formulations by the measurement of the plasma levels of aspartate aminotransferase (AST), alanine aminotransferase (ALT), creatinine, and bilirubin using commercially available kits from Sigma Aldrich, South Africa. The activities of ALT, AST, creatinine, and bilirubin were calculated for each assay using the Equations below:

(3) $$\mathrm{ALT}\;\mathrm{activity}=\frac{B\times \mathrm{Sample}\;\mathrm{Dilution}\;\mathrm{Factor}}{\left(\mathrm{Final}\;\mathrm{temperate}-\mathrm{Initial}\;\mathrm{temperature}\right)\times V\;}$$

where *B* is the amount (nmole) of pyruvate generated between the initial temperature and final temperate, the final temperature is the time of first reading in minutes, the initial temperature is the time of penultimate reading in minutes, and *V* is the sample volume (mL) added to well.

(4) $$\mathrm{AST}\;\mathrm{activity}=\frac{B\times \mathrm{Sample}\;\mathrm{Dilution}\;\mathrm{Factor}}{\left(\mathrm{Reaction}\;\mathrm{Time}\right)\times V\;}$$

where *B* is the amount (nmole) of glutamate generated between the initial temperature and final temperate, the reaction time is the final temperature minus the initial temperature in minutes, and *V* is the sample volume (mL) added to the well.

(5) $$\mathrm{Concentration}\;\mathrm{of}\;\mathrm{creatinine}=\frac{Sa}{Sv\;}$$

where *Sa* is the amount of creatinine in an unknown sample (nmole) from the standard curve and *Sv* is the sample volume (µL) added into the wells, C is the concentration of creatinine in the sample, and the creatinine molecular weight is taken as 113.12 g/mole.

(6) $$\mathrm{Bilirubin}\;\mathrm{concentration}\frac{(A_{530})\;\mathrm{sample}-(A_{530})\;\mathrm{blank}}{(A_{530})\mathrm{calibrator}-(A_{530})\;\mathrm{water}}\times \left(5\;\mathrm{mg}/\mathrm{dL}\right)$$

where (*A*_530_) sample is the value of the sample (total or direct) and (*A*_530_) blank is the value of the sample blank, while (*A*_530_) calibrator and (*A*_530_) water are the value of the calibrator reading and the value of the water control reading, respectively. 5 mg/dL is the equivalent bilirubin concentration of the calibrator.

To determine the toxicity of the SLN formulations to the organ, tissue samples (liver, lung, kidney, and spleen) were obtained and placed in 10% neutral buffered formalin thereafter inserted in paraffin for sectioning the tissues. The tissues were then stained with eosin and hematoxylin for microscopic determination and spotted for toxicity markings.

### 4.16. In Vivo Parasitological Study

#### 4.16.1. Infection of Animals

The in vivo antischistosomal study was conducted in the Schistosome Biological Supply Centre (SBSC) of Theodor Bilharz Research Institute (TBRI), Giza, Egypt. Male Swiss albino mice (CD-1) weighing 18–20 g were obtained from SBSC of TBRI (Giza, Egypt), and housed in an environmentally controlled room temperature of 20–22 °C, a 12 h light/dark cycle, and 50–60% humidity throughout the acclimatization and experimental periods, with access to food and water ad libitum. A hundred microliters of cercarial suspension were gently mixed before being counted and stained with a picric acid solution.

The mice were then infected with *S. mansoni* cercariae (supplied by SBSC) by subcutaneous injection [38] and exposure to 60 ± 10 cercariae per animal. The TBRI Institutional Review Board authorized all of the animal studies, which were carried out in line with the Guide for the Care and Use of Laboratory Animals.

#### 4.16.2. Experimental Design

Mice infection: According to Liang et al. [38] *S. mansoni* cercariae were injected subcutaneously with 60 ± 10 Egyptian strain *S. mansoni* cercariae shedding from *Biomphalariaalexandrina* snails.

The animals were split into five groups and each group comprised ten animals based on the timing of drug administration:

Group 1: Infected control.
Group 2: Single dose 250 mg/kg (PZQ equivalent) CLPF was administered two weeks post-infection.
Group 3: A single dosage of PZQ 250 mg/kg was administered two weeks post-infection.
Group 4: A single dosage of 250 mg/kg of CLPF was administered four weeks post-infection.
Group 5: A single dosage of PZQ 250 mg/kg was administered four weeks post-infection.

After six weeks post-infection, all animals were euthanized.

#### 4.16.3. Assessment of Parasitological Cure Rate

Worm recovery: Euthanized mice were subjected to a hepato-portomesentric perfusion method to collect adult *S. mansoni*, assess sex (male/female/copula), estimate worm load, and then compute the percentage of overall worm reduction [39].

Oogram pattern: The percentage of eggs at various stages of development (oogrampattern) was investigated [40]. The eggs were identified and counted at different stages of development in three intestinal segments, and the mean number of each stage was determined.

Egg count in tissues: Small pieces of intestinal and hepatic tissue were weighed, digested overnight in a 5 mL solution of KOH 5%, and three samples (each 50 μL) of the digested tissue were inspected microscopically to estimate the mean egg count [40]. The method described by Kloetzel [41] was used to determine the number of eggs per gram of tissue and the percentage decrease in total ova per gram of tissue.

### 4.17. Statistical Analysis

The significance of the difference observed was evaluated using a student’s *t*-test, and the findings are presented as mean values and standard deviation (±SD) (GraphPad Prism software, GraphPad prism version 9, Inc., San Diego, CA, USA). *p*-values of less than 0.0001 are considered statistically significant in all tests.

Other data were coded and input using the Microsoft Excel 2010 statistical software. The following calculation was used to compute the percentage of reduction in worm/egg load in each treatment group: [(Number of worms/eggs in the control group) − (Number of worms/eggs in the treated group)] / (Number of worms/eggs in the control group) × 100.

## 5. Conclusions

The development of new antischistosomal agents requires a lot of money and time. Nanoparticulate-based drug delivery systems can be promising to solve the problem of antischistosomal drugs (PZQ) currently in use, which have poor solubility, chemical instability, inadequate bioavailability profile, and rapid metabolism. In this study, PZQ-loaded Compritol lecithin SLNs stabilized with PF127 were successfully prepared using solvent injection co-homogenization techniques. From the in vitro drug release behaviour, it can be deduced that the solvent injection co-homogenization methods of preparing Compritol and lecithin SLNs are suitable for drug delivery application with PF127 as a stabilizer, due to the slow or sustained release of PZQ from SLNs. Furthermore, the addition of PF127 as the surfactants provided the long-term stability of Compritol ATO 888-lecithin solid lipid nanoparticles nanosuspension over 120 days at room temperature. RAW 264.7 macrophages showed acceptable, dose-dependent levels of viability following treatment with SLNs in an in vitro toxicity study. The findings from in vivo toxicity examination corroborated the results obtained in vitro cell culture study, which showed that there is no toxicity or lesser oxidative stress caused by the formulated CLPF-SLNs. The parasitological data showed that a single (250 mg/kg) oral dose of CLPF-SLNs significantly improved the antischistosomal activity of PZQ in *S. mansoni*-infected mice both two and four weeks post-infection. The fabricated CLPF-SLNs demonstrated significant efficiency in the delivery of PZQ; hence, they are promising in drug delivery applications for the treatment of Schistosomiasis.

## Figures and Tables

**Table 1:** Particle size, PDI, the zeta potential of unloaded and PZQ-loaded Compritol-lecithin solid lipid nanoparticles made with PF127, drug entrapment efficacy, and drug loading capacity.

S/N	Sample	Size (nm)	PDI (a.u)	Zeta Potential (mV)	%DEE	%LC
1	CLF-SLN	101.6 ± 0.7	0.27 ± 0.006	−23.6 ± 0.26	-	-
2	CLPF-SLN	112.9 ± 1.0	0.23 ± 0.010	−19.0 ± 0.26	71.63 ± 0.3	11.46 ± 0.61

Note: Values are expressed as mean ± SD (*n* = 3).

**Table 2:** The summary of the DSC thermogram data of all the excipients and the formulated unloaded and PZQ-loaded SLNs.

S/N	Composition	T (Onset)/°C	T (Peak)/°C	ΔH (J/g)
1	PF127 (F)	54.02	55.84	−101.37
2	PZQ (P)	139.60	141.37	−115.10
3	Compritol (C)	69.08	72.29	−124.52
4	Lecithin (L)	222.85	223.99	−1.95
5	Comp-Lec-F127 (CLF)	52.64	55.22	−56.33
6	Comp-Lec-PZQ-F127(CLPF)	52.57	55.38	−65.05

**Table 3:** Effect of PZQ and CLPF (single dose 250 mg/kg two weeks post-infection) on worm loadand their sex in *S. mansoni*-infected mice euthanized six weeks post-infection.

	Mean Worm Burden ± SD (Liver and Porto-Mesenteric)				% Reduction in Total Worm Burden
	Male	Female	Couples	Total	
Control	2.33 ± 0.81	0.33 ± 0.52	6.17 ± 0.75	15.00 ± 0.89	
PZQ	1.33 ± 1.21	0	4.33 ± 0.82	10.00 ± 1.41	33.30
CLPF	1.50 ± 0.55	0	3.67 ± 1.21	8.83 ± 2.64	41.13

**Table 4:** Effect of PZQ and CLPF (single dose 250 mg/kg two weeks post-infection) on number of ova/gm tissues in *S. mansoni*-infected mice euthanized six weeks post-infection.

Mice Group	Liver	% Reduction in Ova Count in Liver	Intestine	% Reduction in Ova Count in the Intestine
Control	28,202 ± 4372		31,902 ± 4342	
PZQ	20,303 ± 2175	28.00	22,702 ± 5347	28.84
CLPF	16,548 ± 6919	41.32	21,120 ± 6644	33.79

**Table 5:** Effect of PZQ and CLPF (single dose 250 mg/kg four weeks post-infection) on worm load and sex in *S. mansoni*-infected mice euthanizedsix weeks post-infection.

	Mean Worm Burden ± SD (Liver and Porto-Mesenteric)				% Reduction in Total Worm Burden
	**Male**	**Female**	**Couples**	**Total**	
Control	2.33 ± 0.81	0.33 ± 0.52	6.17 ± 0.75	15.00 ± 0.89	
PZQ	1.50 ± 1.40	0	2.00 ± 0.89	5.50 ± 2.60	63.30
CLPF	1.00 ± 0.63	0	1.33 ± 1.37	4.33 ± 1.86	71.13

**Table 6:** Effect of PZQ and CLPF (single dose 250 mg/kg four weeks post-infection) on number of ova/gm tissues in *S. mansoni*-infected mice euthanized six weeks post-infection.

Mice Group	Liver	% Reduction in Ova Count in Liver	Intestine	% Reduction in Ova Count in Intestine
Control	28,202 ± 4372		31,902 ± 4342	
PZQ	13,626 ± 2936	51.68	14,658 ± 3699	54.05
CLPF	11,384 ± 4135	59.60	10,310 ± 1080	67.68

## Data Availability

All data related to the study has been included in the manuscript.
